# 205. The increasing trend of penicillin resistance in *Streptococcus suis* clinical isolates during the past 5 years in a tertiary hospital in Thailand

**DOI:** 10.1093/ofid/ofad500.278

**Published:** 2023-11-27

**Authors:** Natthapol Kiatkangwanchon, Kanphai Wongjarit, Sumanee Nilgate, Tanittha Chatsuwan, Chusana Suankratay

**Affiliations:** Chulalongkorn University, Bangkok, Krung Thep, Thailand; Chulalongkorn University, Bangkok, Krung Thep, Thailand; Chulalongkorn University, Bangkok, Krung Thep, Thailand; Chulalongkorn University/Center of Excellence in Antimicrobial Resistance and Stewardship, Bangkok, Krung Thep, Thailand; Chulalongkorn University, Bangkok, Krung Thep, Thailand

## Abstract

**Background:**

*Streptococcus suis*, an emerging zoonotic pathogen, is able to cause a wide spectrum of infections including primary bacteremia, meningitis, endocarditis, and other localized infections. Due to the increasing and widespread use of antibiotics in pig industry in Thailand, will contribute to the emergence of antimicrobial resistance to infection caused by *S. suis*. We aimed to determine the in vitro susceptibility of *S. suis* clinical isolates, in accompanying with the treatment outcomes.

**Methods:**

A retrospective study was carried out on all patients with *S. suis* bacteremia at King Chulalongkorn Memorial Hospital, Bangkok, Thailand, from 2017 to 2022. *S. suis* identification was analyzed by conventional biochemical tests, the API-20 STREP system (bioMérieux, Marcy-l'Étoile, France), or MALDI-TOF MS. In vitro susceptibility test of penicillin, cefotaxime, meropenem, and vancomycin was carried out by E-test according to CLSI recommendation.

**Results:**

There were 32 patients with 17 (53.1%) male and median age of 57 (IQR: 50-65) years. Comorbidities were diabetes (10 patients, 31.3%) and cancer (4, 12.5%). 12 (37.5%) patients had a history of consumption of undercooked pork products. Regarding clinical syndrome, there were 15 (46.9%), 6 (18.8%), 6 (18.8%), and 3 (9.4%) patients with primary bacteremia, infective endocarditis, meningitis, and septic arthritis/spondylodiscitis, respectively. The median length of stay was 13 (IQR: 5-16) days. The most common empirically used antibiotic was ceftriaxone (26 patients, 81.3%), followed by meropenem (3, 9.4%), and ampicillin (2, 6.2%). De-escalation treatment to penicillin G sodium was performed in 11 (34.4%) patients. Of 29 patients with available outcomes, there were 19 (65.5%), 6 (20.7%), and 3 (10.3%) patients with complete recovery, hearing loss, and mortality, respectively.

In vitro susceptibility results are shown in Tables 1 and 2.

**Table 1.**
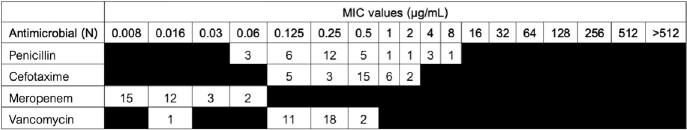
The minimum inhibitory concentration (MIC) distribution for Streptococcus suis blood isolates.

**Table 2.**
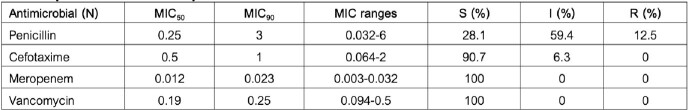
MIC50, MIC90, MIC ranges, and susceptibility according to 2023 CLSI clinical breakpoints for Streptococcus suis blood isolates.

**Conclusion:**

*S. suis* bacteremic patients still had favorable outcomes despite the emergence of penicillin resistance. All isolates were susceptible to meropenem and vancomycin, and most were susceptible to cefotaxime. However, only 28.1% was still susceptible to penicillin. Third-generation cephalosporin should be the first agent for empirical treatment in patients with suspected *S. suis* bacteremia.

**Disclosures:**

**All Authors**: No reported disclosures

